# Association of social determinants of health and age at menopause: NHANES 1999–2018 observational study

**DOI:** 10.1093/hropen/hoaf050

**Published:** 2025-08-18

**Authors:** Yu Guan, Qian Liu, Zhimin Deng, Sirui Liu, Jia Liang, Yujie Zou, Tailang Yin, Dongdong Tang, Jue Liu, Yan Zhang

**Affiliations:** Reproductive Medicine Center, Renmin Hospital of Wuhan University, Wuhan, Hubei, China; Reproductive Medicine Center, Renmin Hospital of Wuhan University, Wuhan, Hubei, China; Reproductive Medicine Center, Renmin Hospital of Wuhan University, Wuhan, Hubei, China; Reproductive Medicine Center, Renmin Hospital of Wuhan University, Wuhan, Hubei, China; Reproductive Medicine Center, Renmin Hospital of Wuhan University, Wuhan, Hubei, China; Reproductive Medicine Center, Renmin Hospital of Wuhan University, Wuhan, Hubei, China; Reproductive Medicine Center, Renmin Hospital of Wuhan University, Wuhan, Hubei, China; Department of Obstetrics and Gynecology, Reproductive Medicine Center, The First Affiliated Hospital of Anhui Medical University, Hefei, China; NHC Key Laboratory of Study on Abnormal Gametes and Reproductive Tract, Anhui Medical University, Hefei, China; Key Laboratory of Population Health Across Life Cycle, Anhui Medical University, Ministry of Education of the People’s Republic of China, Hefei, China; Department of Epidemiology and Biostatistics, School of Public Health, Peking University, Beijing, China; Institute for Global Health and Development, Peking University, Beijing, China; Key Laboratory of Epidemiology of Major Diseases, Ministry of Education, Peking University, Beijing, China; Department of Clinical Laboratory, Institute of Translational Medicine, Renmin Hospital of Wuhan University, Wuhan, Hubei, China

**Keywords:** social determinants of health, menopause, nationwide cross-sectional study, reproductive health, health inequality, National Health and Nutrition Examination Survey

## Abstract

**STUDY QUESTION:**

Do social determinants of health (SDoH) influence the age at menopause among women?

**SUMMARY ANSWER:**

In our study, adverse SDoH, particularly family low income-to-poverty ratio (PIR), low education level, and the marital status of being widowed, are associated with earlier age at menopause.

**WHAT IS KNOWN ALREADY:**

Some prior studies have considered certain SDoH variables (such as educational attainment and marital status) as potential factors influencing age at menopause, but systematic evidence clearly defining the relationship between multidimensional SDoH and menopausal age remains lacking.

**STUDY DESIGN, SIZE, DURATION:**

This cross-sectional analysis included 6083 naturally menopausal women from 10 cycles (1999–2018) of the United States National Health and Nutrition Examination Survey (NHANES) and excluded cases of surgical menopause.

**PARTICIPANTS/MATERIALS, SETTING, METHODS:**

The participants were derived from a nationally representative sample of the NHANES 1999–2018 in the USA. Eight SDoH variables were assessed: employment, PIR, food security, education, healthcare access, health insurance, housing stability, and marital status. Age at menopause was determined by self-reported last menstrual period among women with natural menopause. This study constructed weighted multivariate linear regression models and weighted quantile sum (WQS) analyses and calculated regression coefficients (β) and their 95% CIs. Subgroup analyses and sensitivity analyses were used to verify the robustness of our findings.

**MAIN RESULTS AND THE ROLE OF CHANCE:**

After adjusting for relevant confounding factors, adverse PIR, education level, and marital status (such as being widowed) were significantly associated with earlier age at menopause. Specifically, compared to women with a PIR ≥500%, women with a PIR between 100% and 300% or PIR ≤100% had an earlier age at menopause by 0.877 years (95% CI: −1.526, −0.229, *P* = 0.008) and 1.296 years (95% CI: −2.105, −0.487, *P* = 0.002), respectively. Additionally, compared to women with an educational level of college or above, women with a high school education or less than a high school education had earlier age at menopause by 1.262 years (High school: 95% CI = −1.914, −0.609, *P* < 0.001) and 1.403 years (Less than high school: 95% CI = −2.062, −0.743, *P* < 0.001), respectively. Compared to women who were married or living with a partner, widowed women had earlier age at menopause by 1.363 years (95% CI = −1.887, −0.839, *P* < 0.001). Analysis using a WQS regression model based on decile categorization demonstrated that each 1-unit increase in the composite exposure index of adverse SDoH factors was associated with 3.302 years earlier age at menopause in women (95% CI = −4.129, −2.476, *P* < 0.001). The PIR contributed most substantially to the inverse association between SDoH and age at menopause.

**LIMITATIONS, REASONS FOR CAUTION:**

The cross-sectional design limits causal inference. Unmeasured confounders (e.g. parity, previous hormone use, chemical exposures) and recall bias may persist despite sensitivity analyses.

**WIDER IMPLICATIONS OF THE FINDINGS:**

These findings substantiate the implementation of integrated multidimensional interventions targeting economic stability, housing security, employment support, and healthcare access, which would likely yield substantially greater benefits than single-dimensional policy adjustments. Moreover, material deprivation factors may exert profoundly stronger effects on reproductive aging than previously thought.

**STUDY FUNDING/COMPETING INTEREST(S):**

This work was supported by the National Key Research and Development Program of China (2023YFC2705700), the Interdisciplinary Innovative Talents Foundation from Renmin Hospital of Wuhan University (JCRCYG-2022-009), and the National Natural Science Foundation of China (72474005). All authors declare no competing interests.

**TRIAL REGISTRATION NUMBER:**

N/A.

WHAT DOES THIS MEAN FOR PATIENTS? This study looked at how social and economic factors can affect the age at menopause.

Menopause is a natural part of every woman’s life. It marks the end of her menstrual cycles and her ability to have children. It is also linked to many health issues, such as heart disease, diabetes, and weaker bones. Knowing what factors influence when menopause happens is important for improving women’s long-term health outcomes.

We used data from the National Health and Nutrition Examination Survey (NHANES), collected between 1999 and 2018, to see how factors like job status, family income, education level, and marital status affect a woman’s age at menopause. Our results showed that women with lower income or less education, and those who were widowed tended to go through menopause at an earlier age. In addition, being exposed to multiple negative social and economic conditions was generally linked to an even earlier menopause. Among all the factors we studied, family income had the strongest link to earlier menopause.

## Introduction

Women’s health is a major public health concern, and menopause represents a critical transitional phase in the female lifespan with significant biological and clinical implications. As a natural physiological process, menopause not only marks the cessation of menstrual cycles and the loss of fertility (Dunneram *et al.*, 2019), but is also widely recognized as an important indicator of overall health status and the aging process in women. This process is influenced by a complex interplay of genetic (Zhang *et al.*, 2021), hormonal (Raj *et al.*, 2023), and environmental factors (Levine and Hall, 2023). The age at natural menopause is defined as the age at which a woman naturally transitions into menopause. Abnormal ages at menopause are associated with an increased risk of various health issues. Studies demonstrate that early menopause is significantly linked to an elevated risk of cardiovascular disease, dyslipidemia, osteoporosis, diabetes, and cognitive disorders (Anagnostis *et al.*, 2019; Goh *et al.*, 2019; Liu *et al.*, 2023; Sochocka *et al.*, 2023). Moreover, early menopause not only significantly increases women’s all-cause mortality but may also shorten their life expectancy (Zhang *et al.*, 2019; Xing *et al.*, 2023). Late menopause, on the other hand, has been found to be associated with elevated blood pressure and increased risks of breast cancer (Zhang *et al.*, 2023) and endometrial cancer (Wu *et al.*, 2019). A woman’s reproductive lifespan refers to the duration during which she has the ability to bear children. Among women with a similar age at menarche, the age at menopause plays a critical role in determining the duration of their reproductive lifespan. Existing studies have demonstrated that the length of a woman’s reproductive lifespan is closely linked to her health status, including pulmonary dysfunction, particularly restrictive ventilatory disorders (Lim *et al.*, 2020), cardiovascular diseases or events (Chen *et al.*, 2022; Hu *et al.*, 2022), neurological disorders such as Parkinson’s disease (Nitkowska *et al.*, 2014) and chronic kidney disease (Kang *et al.*, 2020; Rytz *et al.*, 2022). Therefore, deepening the understanding of women’s age at menopause holds important value for exploring fertility, healthy aging, and disease risk. It also provides scientific evidence for the development and improvement of women’s health management and disease prevention strategies.

Social determinants of health (SDoH) constitute a multidimensional comprehensive evaluation system that includes environmental factors related to where individuals are born, grow, live, work, and age, profoundly influencing health inequities and health status differences within populations (Dobson *et al.*, 2022). These factors encompass housing conditions, community poverty levels, food security, access to green spaces, transportation convenience, racial background, and various psychosocial stressors, all of which collectively influence individual health behavior patterns (Chaturvedi *et al.*, 2024). Many scholars argue that SDoH may have a greater impact on health compared to healthcare services or personal lifestyle choices (Dobson *et al.*, 2022). As global attention to health inequities continues to rise, researchers are increasingly exploring how these social factors specifically affect women’s reproductive health. For instance, Namazi *et al.* (2019) emphasized in their comprehensive review that measures such as improving educational opportunities, enhancing emotional and social support, optimizing lifestyle, and elevating socioeconomic status can contribute to better menopausal health outcomes for women. Although some studies have included certain SDoH variables (such as educational level and marital status) as potential influencing factors when examining menopause age (Alinia *et al.*, 2023; Gonzalez-Martin *et al.*, 2023), there remains a lack of systematic evidence clearly defining the relationships between various SDoH and these reproductive health indicators. Additionally, the current research is relatively limited in elucidating how different SDoH affect women’s age at menopause, as well as the racial disparities reflected in representative samples and the primary SDoH and their mechanisms of action.

This study aims to utilize national cross-sectional data collected from the United States National Health and Nutrition Examination Survey (NHANES) between 1999 and 2018 to conduct an in-depth investigation into the associations between SDoH and women’s age at menopause, thereby addressing gaps in the existing research field. Through this study, we hope to provide new insights into how social and environmental factors influence women’s reproductive health and to offer robust scientific support for the formulation of policies aimed at reducing health inequities.

## Materials and methods

### Study population

NHANES, a long-term cross-sectional survey project, aims to comprehensively assess the health and nutritional status of the US population. NHANES has been employing a complex, stratified, multi-stage probability sampling method every 2 years since 1999–2000 to select a nationally representative sample of non-institutionalized US civilians. The data collected through NHANES cover various aspects, including demographic characteristics, physical examinations, laboratory test results, and questionnaire interviews. Detailed information about the project can be accessed on the NHANES official website (https://www.cdc.gov/nchs/nhanes/index.htm). Additionally, the NHANES study protocol has been approved by the National Center for Health Statistics Institutional Review Board, and all participants provided written informed consent after being fully informed about the study content.

**Figure 1.**
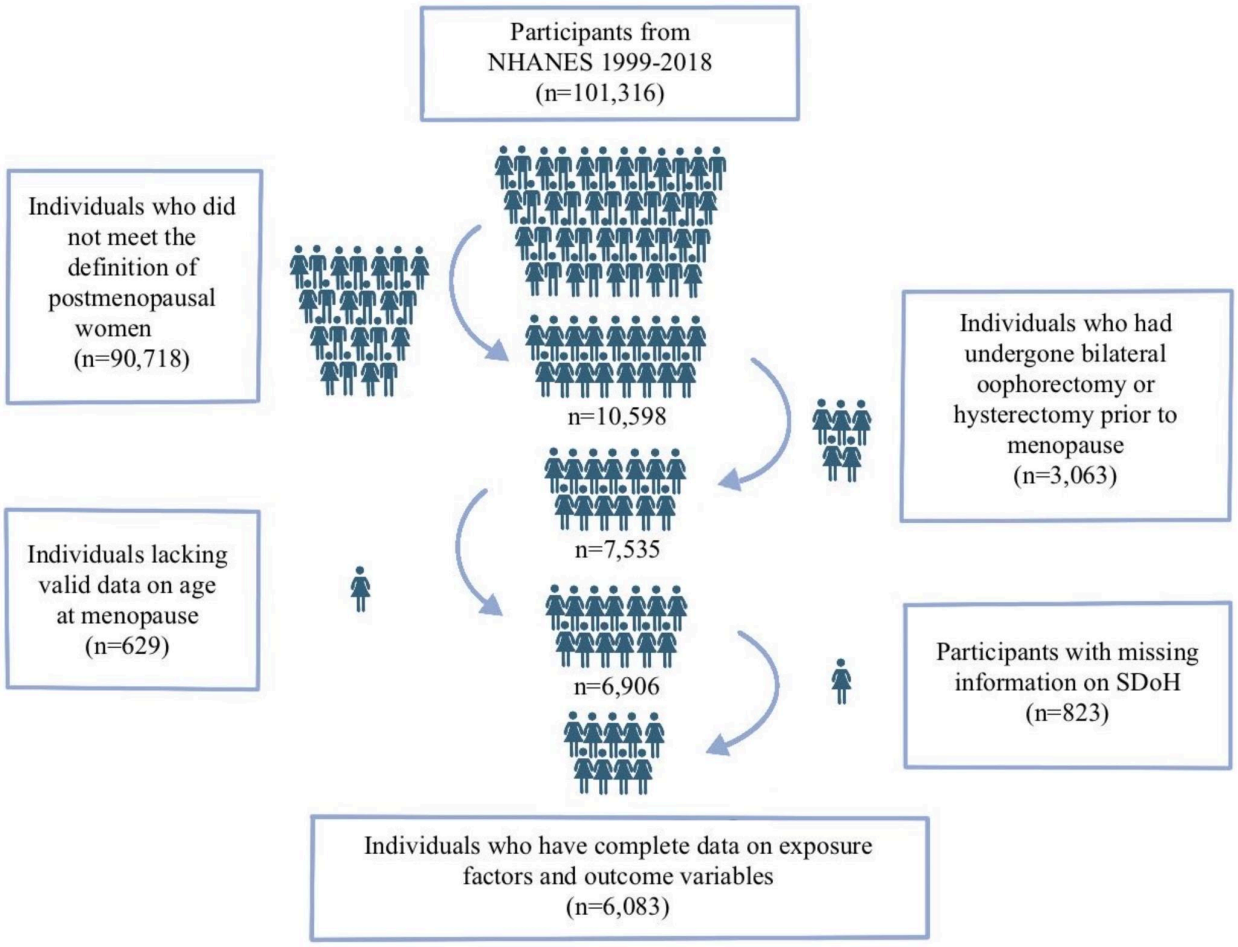
**Flowchart illustrating the enrollment process of participants in US NHANES 1999–2018.** NHANES, National Health and Nutrition Examination Survey; SDoH, social determinants of health. Figure was created using Procreate (version 5.2.2, Savage Interactive Pty Ltd, Hobart, Australia).

This cross-sectional study encompasses 10 survey cycles of NHANES from 1999 to 2018. Initially, we identified postmenopausal women (defined as those who reported cessation of regular menstruation due to menopause in the past 12 months) from the overall sample (n = 101 316), yielding a preliminary sample of 10 598 women. Subsequently, we excluded women who had undergone bilateral oophorectomy or hysterectomy prior to menopause (n = 3063), those lacking valid data on age at menopause (n = 629), and participants with missing information on SDoH (such as employment status and family income-to-poverty ratio (PIR)) (n = 823). After these screening steps, the final analytical sample consisted of 6083 participants with complete data on all exposure factors and outcome variables for subsequent statistical analysis (see Fig. 1).

### Determination of SDoH

Data on SDoH were derived from the questionnaire section of the NHANES survey. The overall framework and specific variable classifications were based on previous relevant studies (Bundy *et al.*, 2023; Liang *et al.*, 2024) and aligned with the five domains of SDoH outlined in ‘Healthy People 2030’ (economic stability, education access and quality, health care access and quality, neighborhood and built environment, and social and community context). This study ultimately focused on eight available SDoH variables: (i) employment status; (ii) family PIR; (iii) food security; (iv) education level; (v) regular health care access; (vi) cover by health insurance; (vii) housing instability; and (viii) marital status. Each variable was categorized based on its condition, ranging from favorable to unfavorable. In principle, the most favorable category served as the reference group. Unless otherwise specified, comparisons mentioned in the text default to the most favorable variable category, to illustrate changes in outcomes among groups under less favorable SDoH conditions. For detailed descriptions of SDoH-related questions, please refer to Supplementary Table S1.

### Determination of age at menopause

Data on reproductive outcomes in this study were sourced from the NHANES Reproductive Health Questionnaire (RHQ). All questionnaire items were administered through a Computer-Assisted Personal Interviewing system. Trained interviewers had conducted face-to-face interviews with participants at the NHANES Mobile Examination Centers, ensuring high accuracy and reliability of the data. Specifically, age at menopause had been determined by asking naturally menopausal women about their age at the last menstrual period (RHQ060). Natural menopause was defined as the absence of regular menstrual periods for the past 12 months due to menopause, with no history of bilateral oophorectomy or hysterectomy before menopause. Relevant questions included RHQ040/RHD042/RHD043, RHQ310/RHQ305, RHD280, RHQ290/RHQ291, and RHQ330/RHQ340/RHQ332. Individuals who reported undergoing bilateral oophorectomy or hysterectomy with the procedure performed before menopause were excluded from the study. The remaining participants, including those who: (i) did not undergo these procedures, (ii) responded ‘refused’ or ‘don’t know’, (iii) had missing surgical history data, or (iv) underwent the procedures after menopause, were all included in the study cohort. We also defined early menopause as  < 48 years and late menopause as >54 years, with 48–54 years considered normal, consistent with previous studies (Gonzalez-Martin *et al.*, 2023).

### Covariates

Based on previous studies (Alinia *et al.*, 2023; Lin *et al.*, 2024), we selected the following potential confounding variables from demographic and questionnaire data for our analysis: age, race/ethnicity, smoking status, alcohol consumption, and chronic comorbidities. Age was categorized into three groups: ≤59 years, 60–70 years, and ≥70 years. Race/ethnicity was classified according to NHANES standard categories, including Mexican American, Non-Hispanic White, Non-Hispanic Black, Other Hispanic, and Other Race. Smoking status was categorized based on the questionnaire item SMQ020: ‘Have you smoked at least 100 cigarettes in your entire life?’ Those who answered ‘Yes’ were classified as smokers, and the rest as non-smokers. Alcohol consumption was categorized based on the question ‘In the past 12 months, did you drink at least 12 drinks of any alcoholic beverage’, with responses classified as ‘Yes’ or ‘No’, corresponding to questionnaire items ALQ100, ALD100, ALQ101, and ALQ121. Chronic comorbidities refer to conditions that may affect reproductive health, including hypertension, diabetes, coronary heart disease, heart failure, or stroke. These were collected through self-reporting, specifically the question ‘Has a doctor or other health professional ever told you that you had this condition?’ corresponding to questionnaire items BPQ020, DIQ010, MCQ160C, MCQ160B, and MCQ160F. If an individual had been diagnosed with any of these conditions, they were marked as ‘Yes’; otherwise, they were marked as ‘No’.

### Statistical analysis

To address the complex sampling design of NHANES, all statistical analyses were weighted. We followed the NHANES data processing guidelines and used the interview weights WTINT2YR (for data from 1999 to 2002, WTINT4YR was used). First, we described the basic characteristics of the NHANES sample included in the study. Continuous variables were presented using mean±SD and analyzed using weighted *t*-tests or Kruskal–Wallis *H* tests. Categorical variables were presented as frequencies and percentages and analyzed using chi-square tests. We assessed differences in baseline characteristics of participants based on age at menopause ( < 48 years, 48–54 years, >54 years) (Gonzalez-Martin *et al.*, 2023). Next, we performed Spearman correlation analyses among the eight SDoH variables to explore potential multicollinearity issues. To investigate the relationships between the eight SDoH variables and age at menopause, we constructed weighted multivariate linear regression models and calculated regression coefficients (β) and their 95% CIs. The models were set up as follows: Crude model: unadjusted for any covariates. Model 1: adjusted for age and race/ethnicity. Model 2: further adjusted for age, race/ethnicity, and the other seven categorical SDoH variables. Given established evidence demonstrating possible racial/ethnic variations in age at menopause (Henderson *et al.*, 2008; Nowakowski and Graves, 2017; Reeves *et al.*, 2023), and recognizing that these differences may be intricately associated with SDoH such as socioeconomic status and healthcare access, we adjusted for race/ethnicity as a covariate in Model 1 and Model 2. Concurrently, we acknowledge that race is itself frequently conceptualized as an SDoH proxy variable in contemporary research (Zhong *et al.*, 2024). Considering that race/ethnicity may inherently reflect systemic discrimination and structural inequities (Egede *et al.*, 2024) (e.g. disparities in healthcare resource allocation), adjusting for race/ethnicity could potentially lead to over-adjustment bias, thereby attenuating the true effects of other SDoH factors. To address this methodological consideration, we specifically constructed Model 3 which maintains adjustment for all other covariates while deliberately excluding race/ethnicity, enabling comparative analysis of SDoH effects under different adjustment frameworks. In all four models, we also conducted trend tests using linear regression. Additionally, we performed subgroup analyses to explore whether the relationships between SDoH variables and outcome measures varied by age, race/ethnicity, and survey cycle. To verify the robustness of our findings, we conducted sensitivity analyses in Model 2 by differentially adjusting for smoking status, alcohol consumption, and chronic comorbidities in various combinations.

Finally, to evaluate the overall effect of multiple SDoH variables and identify specific SDoH variables that play a key role in the combined effect, we conducted weighted quantile sum (WQS) analysis, adjusting for age, race/ethnicity, smoking status, and alcohol consumption. WQS analysis is a multivariate regression statistical model suitable for high-dimensional datasets. It constructs a weighted index to test the overall impact of a set of exposure variables on the outcome. Additionally, this model assesses the relative importance of each variable in affecting the outcome by assigning weights to each exposure variable. In this study, we constructed WQS indices based on the deciles of the SDoH variables and divided the dataset into a training set (40%) and a validation set (60%), conducting 100 Bootstrap samplings. All statistical analyses were performed using R statistical software (version 4.4.1; R Foundation for Statistical Computing, Vienna, Austria). The significance level for statistical tests was set at a two-sided *P* < 0.05.

## Results

### Baseline characteristics of study participants

**Figure 2.**
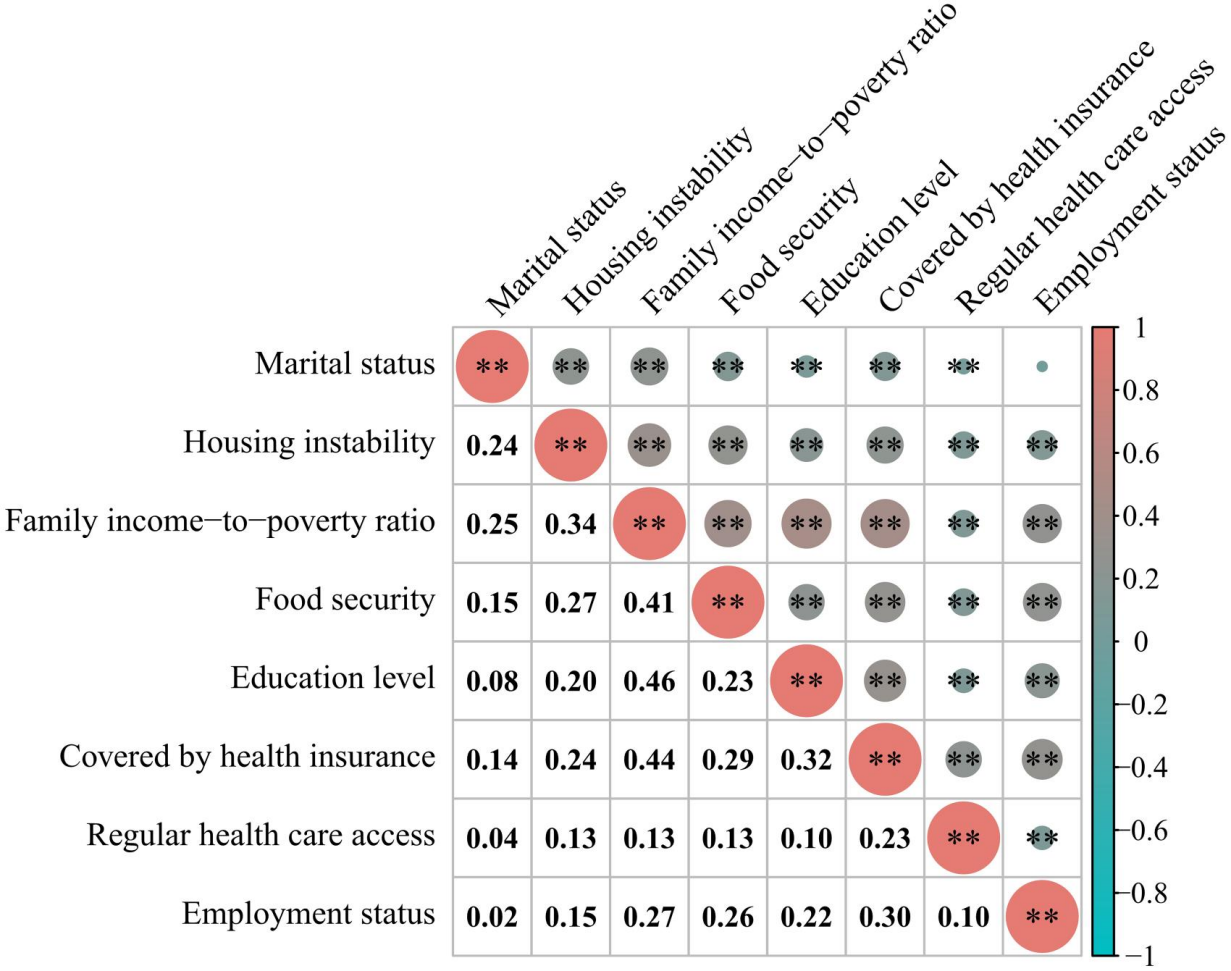
**Spearman correlations between eight SDoH variables in US NHANES 1999–2018 (n = 6083).** Pink to blue: Correlation coefficients (ρ) from +1 (strong positive, pink) to −1 (strong negative, blue); *P < 0.05, **P < 0.01; Numeric values show Spearman’s ρ (e.g. 0.24 = weak positive correlation). Larger absolute values indicate stronger associations (e.g. 0.46). NHANES, National Health and Nutrition Examination Survey; SDoH, social determinants of health.

In this study, data from 6083 participants in the NHANES database were included (mean age: 63.1 ± 9.9 years). Among these participants, 76.5% were non-Hispanic White, 53.1% had chronic conditions that affected reproductive health, 32% had experienced early menopause, and 16% had experienced late menopause (see Table 1). Significant differences were observed in age, race/ethnicity, employment status, family PIR, food security, educational level, health insurance status, housing instability, marital status, smoking status, and chronic comorbidities across groups stratified by age at menopause. Findings revealed that women with later age at menopause were generally older, more likely to be non-Hispanic White, had more stable employment, and exhibited more favorable conditions across most SDoH variables, whereas those with earlier menopause were more likely to smoke and less likely to have chronic comorbidities. The results of the Spearman correlation analysis showed mild to moderate correlations among the eight SDoH variables (Fig. 2).

**Table 1.** Survey-weighted characteristic variables of the study participants stratified by age at menopause, US NHANES 1999–2018 (n = 6083).

Variable	**Overall**, N = 6083 (100%)^a^	Age at menopause			***P*** ^b^
		**Premature, <48 years**, N = 2114 (32%)^a^	**Normal, 48–54 years**, N = 3008 (52%)^a^	**Delayed, >54 years**, N = 961 (16%)^a^	
**Age, years**	63.1 (9.9)	61.6 (11.4)	62.8 (9.1)	67.3 (7.6)	**<0.001** ***
**Race/ethnicity**					**<0.001** ***
* Mexican American*	985 (4.8%)	391 (6.2%)	484 (4.5%)	110 (2.7%)	
* Other Hispanic*	578 (4.5%)	221 (5.3%)	295 (4.8%)	62 (2.1%)	
* Non-Hispanic White*	2952 (76.5%)	958 (72.6%)	1463 (77.3%)	531 (81.6%)	
* Non-Hispanic Black*	1077 (8.5%)	414 (10.6%)	487 (7.5%)	176 (7.8%)	
* Other Race*	491 (5.8%)	130 (5.4%)	279 (6.0%)	82 (5.8%)	
**Employment status**					**<0.001** ***
* Employed, student, retired*	4518 (79.6%)	1464 (73.9%)	2295 (81.8%)	759 (84.1%)	
* Not employed*	1565 (20.4%)	650 (26.1%)	713 (18.2%)	202 (15.9%)	
**Family PIR**					**<0.001** ***
* 500% ≤ PIR*	1106 (28.7%)	319 (24.3%)	603 (30.7%)	184 (31.0%)	
* 300% ≤ PIR < 500%*	1058 (22.5%)	308 (20.3%)	564 (23.5%)	186 (23.7%)	
* 100% < PIR < 300%*	2694 (37.0%)	971 (39.6%)	1290 (35.5%)	433 (36.6%)	
* PIR ≤ 100%*	1225 (11.9%)	516 (15.8%)	551 (10.3%)	158 (8.8%)	
**Food security**					**<0.001** ***
* Full food security (0 affirmative)*	4583 (83.8%)	1506 (79.6%)	2311 (85.6%)	766 (86.5%)	
* Marginal (1–2 affirmative)*	609 (6.6%)	218 (7.1%)	304 (6.4%)	87 (6.1%)	
* Low (3–5 affirmative)*	518 (5.1%)	222 (6.9%)	234 (4.4%)	62 (3.7%)	
* Very low (6–10 affirmative)*	373 (4.5%)	168 (6.4%)	159 (3.5%)	46 (3.7%)	
**Education level**					**<0.001** ***
* College*	1193 (28.1%)	321 (23.0%)	666 (30.8%)	206 (29.4%)	
* Some college*	1589 (28.6%)	518 (27.1%)	800 (28.7%)	271 (30.9%)	
* High school*	1464 (26.0%)	540 (28.5%)	707 (25.3%)	217 (23.2%)	
* Less than high school*	1837 (17.4%)	735 (21.3%)	835 (15.2%)	267 (16.4%)	
**Regular health care access**					0.5
* Routine place for healthcare*	5560 (93.4%)	1907 (93.2%)	2751 (93.2%)	902 (94.6%)	
* No routine place, or ER/hospital/other*	523 (6.6%)	207 (6.8%)	257 (6.8%)	59 (5.4%)	
**Covered by health insurance**					**<0.001** ***
* Private insurance*	3186 (64.6%)	1024 (60.1%)	1647 (67.2%)	515 (65.4%)	
* Government insurance*	2120 (26.5%)	762 (28.9%)	988 (23.9%)	370 (29.7%)	
* No insurance*	777 (8.9%)	328 (11.0%)	373 (8.8%)	76 (4.9%)	
**Housing instability**					**<0.001** ***
* Own home*	4423 (80.7%)	1425 (75.2%)	2241 (82.8%)	757 (85.3%)	
* Rent home*	1523 (17.6%)	643 (23.1%)	690 (15.4%)	190 (13.5%)	
* Other arrangement*	137 (1.7%)	46 (1.7%)	77 (1.8%)	14 (1.2%)	
**Marital status**					**0.007** **
* Married or living with a partner*	2971 (57.1%)	968 (53.5%)	1528 (58.7%)	475 (59.0%)	
* Never married*	402 (5.5%)	147 (6.2%)	205 (5.5%)	50 (3.9%)	
* Widowed*	1467 (18.3%)	530 (20.0%)	673 (16.7%)	264 (20.1%)	
* Divorced*	999 (16.8%)	373 (17.3%)	482 (16.8%)	144 (15.6%)	
* Separated*	244 (2.4%)	96 (3.1%)	120 (2.3%)	28 (1.4%)	
**Year**					0.3
* 1999–2000*	408 (5.6%)	143 (6.3%)	194 (5.3%)	71 (5.1%)	
* 2001–2002*	509 (7.9%)	174 (8.2%)	258 (8.2%)	77 (6.2%)	
* 2003–2004*	567 (8.3%)	209 (9.4%)	255 (7.3%)	103 (9.0%)	
* 2005–2006*	494 (9.2%)	157 (8.4%)	267 (10.1%)	70 (8.1%)	
* 2007–2008*	704 (9.8%)	274 (11.5%)	324 (8.7%)	106 (9.8%)	
* 2009–2010*	731 (11.1%)	270 (11.1%)	347 (11.2%)	114 (10.7%)	
* 2011–2012*	628 (10.9%)	201 (9.6%)	330 (11.8%)	97 (11.1%)	
* 2013–2014*	694 (11.6%)	243 (11.5%)	347 (11.6%)	104 (11.5%)	
* 2015–2016*	671 (12.9%)	236 (12.3%)	324 (12.5%)	111 (15.3%)	
* 2017–2018*	677 (12.7%)	207 (11.7%)	362 (13.3%)	108 (13.1%)	
**Smoking**					**<0.001** ***
* Yes*	2397 (42.8%)	928 (49.7%)	1113 (39.6%)	356 (39.2%)	
* No*	3686 (57.2%)	1186 (50.3%)	1895 (60.4%)	605 (60.8%)	
**Alcohol consumption**					0.13
* Yes*	3015 (58.7%)	1017 (56.1%)	1520 (59.9%)	478 (60.3%)	
* No*	3068 (41.3%)	1097 (43.9%)	1488 (40.1%)	483 (39.7%)	
**Comorbidities**					**<0.001** ***
* Yes*	3622 (53.1%)	1224 (50.7%)	1765 (51.9%)	633 (61.7%)	
* No*	2461 (46.9%)	890 (49.3%)	1243 (48.1%)	328 (38.3%)	

Bold values indicate statistical significance (*P* < 0.05 or *P* < 0.001).

a Mean (SD) for continuous; counting (n) and survey-weighted percentage (%) for categorical.

b The *P* were assessed by design-based Kruskal–Wallis test or Pearson’s χ^2^ test (Rao and Scott adjustment); Asterisks(*) are used to indicate statistical significance levels.

* *P* < 0.05.

** *P* < 0.01.

*** *P* < 0.001.

NHANES, National Health and Nutrition Examination Survey; PIR, family income-to-poverty ratio.

### Associations between SDOH and age at menopause

In this population, we constructed four weighted multivariate linear regression models to explore the associations between SDoH and age at menopause (see Table 2). In the crude model and in Model 1, all seven SDoH variables except regular health care access were significantly negatively associated with age at menopause. In the fully adjusted model (Model 2), PIR, educational level, and marital status remained significantly associated with age at menopause. Specifically, compared to women with a PIR ≥500%, women with a PIR between 100% and 300% and PIR ≤100% had earlier ages at menopause (100% < PIR < 300%: β = −0.877, 95% CI = −1.526, −0.229, *P = * 0.008; PIR ≤100%: β = −1.296, 95% CI = −2.105, −0.487, *P = * 0.002). Compared to women with an educational level of college or above, women with a high school education and less than a high school education had earlier ages at menopause (High school: β = −1.262, 95% CI = −1.914, −0.609, *P < * 0.001; Less than high school: β = −1.403, 95% CI = −2.062, −0.743, *P* < 0.001). Compared to women who were married or living with a partner, widowed women had earlier ages at menopause (β = −1.363, 95% CI = −1.887, −0.839, *P < * 0.001). Additionally, a linear trend association was observed for lower PIR (trend *P* < 0.001) and lower educational level (trend *P* < 0.001) in relation to age at menopause. In Model 3 without adjustment for race/ethnicity, the effect estimates of key SDoH showed minimal numerical differences compared to Model 2 [e.g. 100% < PIR < 300% β changed from −0.877 (95% CI: −1.526, −0.229) to −0.882 (−1.532, −0.233); high school education β changed from −1.262 (−1.914, −0.609) to −1.284 (−1.932, −0.635)], while maintaining identical patterns of statistical significance (for statistically significant SDoH variables, the *P* remained largely unchanged or showed minimal variations). This finding suggests that the SDoH variables in our model may have already accounted for variance potentially attributable to racial/ethnic factors, indicating no substantial additional confounding by race/ethnicity in this context.

**Table 2.** Association between SDoH and age at menopause in survey-weighted linear regression models, US NHANES 1999–2018 (n = 6083).

SDoH variables	Crude Model		Model 1		Model 2		Model 3	
	β(95% CI)	***P*** ^a^	β(95% CI)	***P*** ^a^	β(95% CI)	***P*** ^a^	β(95% CI)	***P*** ^a^
**Employment status**								
* Employed, student, retired*	ref		ref		ref		ref	
* Not employed*	−1.477(−1.989, −0.966)	**<0.001** ***	−0.960(−1.487, −0.433)	**<0.001** ***	−0.394(−0.935, 0.147)	0.152	−0.391(−0.934, 0.151)	0.156
* P for trend*		**<0.001** ***		**<0.001** ***		0.152		0.156
**Family PIR**								
* 500% ≤ PIR*	ref		ref		ref		ref	
* 300% ≤ PIR < 500%*	−0.156(−0.713, 0.401)	0.581	−0.367(−0.918, 0.184)	0.191	−0.049(−0.590, 0.492)	0.859	−0.052(−0.592, 0.488)	0.850
* 100% < PIR < 300%*	−0.998(−1.549, −0.448)	**<0.001** ***	−1.584(−2.173, −0.994)	**<0.001** ***	−0.877(−1.526, −0.229)	**0.008** **	−0.882(−1.532, −0.233)	**0.008** **
* PIR ≤ 100%*	−2.238(−2.844, −1.631)	**<0.001** ***	−2.409(−3.030, −1.788)	**<0.001** ***	−1.296(−2.105, −0.487)	**0.002** **	−1.307(−2.121, −0.493)	**0.002** **
* P for trend*		**<0.001** ***		**<0.001** ***		**<0.001** ***		**<0.001** ***
**Food security**								
* Full food security (0 affirmative)*	ref		ref		ref		ref	
* Marginal (1–2 affirmative)*	−0.652(−1.450, 0.147)	0.109	−0.255(−1.066, 0.555)	0.535	−0.557(−0.303, 1.416)	0.203	−0.521(−0.328, 1.369)	0.227
* Low (3–5 affirmative)*	−1.860(−2.591, −1.128)	**<0.001** ***	−1.227(−1.961, −0.494)	**0.001** **	−0.112(−0.844, 0.620)	0.762	−0.157(−0.893, 0.579)	0.674
* Very low (6–10 affirmative)*	−2.208(−3.248, −1.167)	**<0.001** ***	−1.547(−2.551, −0.542)	**0.003** **	−0.348(−1.445, 0.749)	0.531	−0.374(−1.466, 0.717)	0.499
* P for trend*		**<0.001** ***		**<0.001** ***		0.307		0.282
**Education level**								
* College*	ref		ref		ref		ref	
* Some college*	−0.552(−1.075, −0.028)	**0.039** *	−0.739(−1.258, −0.220)	**0.006** **	−0.412(−0.916, 0.091)	0.108	−0.432(−0.932, 0.068)	0.090
* High school*	−1.383(−2.031, −0.735)	**<0.001** ***	−1.714(−2.350, −1.078)	**<0.001** ***	−1.262(−1.914, −0.609)	**<0.001** ***	−1.284(−1.932, −0.635)	**<0.001** ***
* Less than high school*	−1.838(−2.423, −1.253)	**<0.001** ***	−2.271(−2.886, −1.655)	**<0.001** ***	−1.403(−2.062, −0.743)	**<0.001** ***	−1.468(−2.111, −0.825)	**<0.001** ***
* P for trend*		**<0.001** ***		**<0.001** ***		**<0.001** ***		**<0.001** ***
**Regular health care access**								
* Routine place for healthcare*	ref		ref		ref		ref	
* No routine place, or ER/hospital/other*	−0.332(−1.056, 0.391)	0.365	−0.180(−0.521, 0.881)	0.612	−0.655(−0.119, 1.429)	0.096	−0.660(−0.109, 1.429)	0.092
* P for trend*		0.365		0.612		0.096		0.092
**Covered by health insurance**								
* Private insurance*	ref		ref		ref		ref	
* Government insurance*	−0.508(−1.011, −0.004)	**0.048** *	−0.937(−1.452, −0.423)	**<0.001** ***	−0.202(−0.706, 0.302)	0.429	−0.192(−0.698, 0.314)	0.455
* No insurance*	−1.198(−1.750, −0.647)	**<0.001** ***	−0.387(−0.930, 0.157)	0.162	−0.582(−0.015, 1.180)	0.056	−0.554(−0.048, 1.156)	0.071
* P for trend*		**<0.001** ***		0.162		0.056		0.071
**Housing instability**								
* Own home*	ref		ref		ref		ref	
* Rent home*	−1.513(−2.026, −1.000)	**<0.001** ***	−1.260(−1.778, −0.741)	**<0.001** ***	−0.503(−1.088, 0.081)	0.091	−0.489(−1.064, 0.086)	0.095
* Other arrangement*	−0.628(−2.062, 0.806)	0.388	−0.732(−2.117, 0.652)	0.297	−0.176(−1.535, 1.183)	0.798	−0.132(−1.494, 1.230)	0.848
* P for trend*		0.388		0.297		0.798		0.848
**Marital status**								
* Married or living with a partner*	ref		ref		ref		ref	
* Never married*	−1.229(−1.997, −0.462)	**0.002** **	−0.683(−1.402, 0.037)	0.063	−0.484(−1.215, 0.247)	0.193	−0.536(−1.263, 0.191)	0.147
* Widowed*	−0.476(−0.932, −0.021)	**0.041** *	−1.863(−2.400, −1.327)	**<0.001** ***	−1.363(−1.887, −0.839)	**<0.001** ***	−1.387(−1.910, −0.863)	**<0.001** ***
* Divorced*	−0.432(−1.010, 0.146)	0.142	−0.242(−0.790, 0.305)	0.383	−0.001(−0.553, 0.552)	0.999	−0.020(−0.567, 0.527)	0.942
* Separated*	−1.839(−3.109, −0.569)	**0.005** **	−0.934(−2.147, 0.278)	0.130	−0.237(−1.343, 0.870)	0.673	−0.336(−1.437, 0.766)	0.548
* P for trend*		**0.037** *		0.278		0.994		0.898

For each of eight SDoH variables: Crude model was an unadjusted model. Model 1 was adjusted for age and race/ethnicity. Model 2 was adjusted for age, race/ethnicity, and other seven SDoH variables. Model 3 was adjusted for age and other seven SDoH variables. The results show the adjusted regression coefficients (β), their 95% CIs, and *P*. Bold values indicate statistical significance (*P* < 0.05 or *P* < 0.001). *P* for trend indicates the statistical significance of the trend across categories of the SDoH variables.

a Asterisks(*) are used to indicate statistical significance levels.

* *P* < 0.05.

** *P* < 0.01.

*** *P* < 0.001.

NHANES, National Health and Nutrition Examination Survey; PIR, family income-to-poverty ratio; SDoH, social determinants of health.

### Subgroup and sensitivity analyses

We conducted further subgroup analyses on the SDoH variables that showed significant associations in the fully adjusted model (Model 2), stratifying by age, race/ethnicity, and survey cycle (see Table 3, Supplementary Tables S2 and S3). We adjusted for all covariates in Model 2 except the corresponding stratification variables in all subgroup analyses (survey cycle stratification adjusted for all covariates). In the subgroup of women aged 60–70 years, compared to those with college education, those with a high school education (β = −1.736, 95% CI = −2.636, −0.836, *P* < 0.001) and those with less than a high school education (β = −2.014, 95% CI = −3.201, −0.828, *P* = 0.001) had earlier ages at menopause. In addition, compared with women who were married or living with a partner, never-married women (β = −1.168, 95% CI: −2.334, −0.001, *P* = 0.050) and widowed women (β = −1.142, 95% CI: −2.041, −0.242, *P* = 0.013) had earlier ages at menopause. Among women aged >70 years, our analyses demonstrated that those with a PIR ≤100% (β = −1.667, 95% CI: −3.277, −0.057, *P* = 0.042) and separated women (β = −2.758, 95% CI: −4.389, −1.128, *P* = 0.001) experienced a statistically significant earlier ages at menopause. However, for women aged  < 60 years, this study found no significant associations between SDoH factors (including PIR, educational level, and marital status) and age at menopause.

**Table 3.** Associations between SDoH and age at menopause: survey-weighted linear regression models stratified by age, US NHANES 1999–2018 (n = 6083).

SDoH variables	Age <60 (n = 1912)		60≤Age ≤ 70 (n = 2300)		Age >70 (n = 1871)	
	β(95% CI)	***P*** ^a^	β(95% CI)	***P*** ^a^	β(95% CI)	***P*** ^a^
**Family PIR**						
* 500% ≤ PIR*	ref		ref		ref	
* 300% ≤ PIR < 500%*	0.088(−0.814, 0.990)	0.847	−0.233(−1.125, 0.659)	0.607	0.474(−0.725, 1.674)	0.436
* 100% < PIR < 300%*	−0.582(−1.714, 0.549)	0.311	−0.573(−1.543, 0.397)	0.244	−0.158(−1.266, 0.950)	0.778
* PIR ≤ 100%*	−0.399(−1.844, 1.046)	0.586	−0.375(−1.661, 0.911)	0.565	−1.667(−3.277, −0.057)	**0.042** *
**Education level**						
* College*	ref		ref		ref	
* Some college*	−0.238(−1.101, 0.625)	0.586	−0.704(−1.500, 0.092)	0.082	0.090(−0.874, 1.054)	0.853
* High school*	−0.917(−2.084, 0.250)	0.123	−1.736(−2.636, −0.836)	**<0.001** ***	−0.542(−1.575, 0.491)	0.301
* Less than high school*	−1.052(−2.278, 0.174)	0.092	−2.014(−3.201, −0.828)	**0.001** **	−0.193(−1.269, 0.884)	0.724
**Marital status**						
* Married or living with a partner*	ref		ref		ref	
* Never married*	−0.547(−1.684, 0.589)	0.342	−1.168(−2.334, −0.001)	**0.050** *	0.720(−1.430, 2.871)	0.509
* Widowed*	0.289(−0.994, 1.572)	0.657	−1.142(−2.041, −0.242)	**0.013** *	−0.267(−1.033, 0.499)	0.491
* Divorced*	−0.238(−1.196, 0.720)	0.624	−0.074(−0.935, 0.788)	0.866	0.550(−0.867, 1.968)	0.444
* Separated*	−0.038(−1.666, 1.591)	0.963	−0.259(−1.787, 1.268)	0.738	−2.758(−4.389, −1.128)	**0.001** **

For each SDoH variable within the age subgroups, the polynomial linear model was adjusted for race/ethnicity and the other seven SDoH variables. The results show the adjusted regression coefficients (β), their 95% CIs, and *P*. Bold values indicate statistical significance (*P* < 0.05 or *P* < 0.001).

a Asterisks(*) are used to indicate statistical significance levels.

* *P* < 0.05.

** *P* < 0.01.

*** *P* < 0.001.

NHANES, National Health and Nutrition Examination Survey; PIR, family income-to-poverty ratio; SDoH, social determinants of health.

When conducting subgroup analyses by race/ethnicity, we combined ‘other Hispanic’ and other races into the same category. Stratified by race/ethnicity, we observed significant associations of PIR (100% < PIR < 300%: β = −0.891, 95% CI = −1.649, −0.134, *P* = 0.021; PIR ≤100%: β = −1.736, 95% CI = −2.818, −0.653, *P* = 0.002), educational level (High school: β = −1.472, 95% CI = −2.236, −0.708, *P < * 0.001; Less than high school: β = −1.699, 95% CI = −2.565, −0.833, *P* < 0.001), and marital status (Widowed: β = −1.422, 95% CI = −2.040, −0.803, *P* < 0.001) with age at menopause among non-Hispanic White women. Among non-Hispanic Black women, only educational level showed a significant association with age at menopause (Some college: β = −1.236, 95% CI = −2.369, −0.102, *P* = 0.033; Less than high school: β = −1.634, 95% CI = −3.024, −0.245, *P* = 0.022). Overall, the main positive findings were mostly concentrated among non-Hispanic White women, and the direction of the associations was consistent with our previous findings. However, no significant associations between these SDoH factors and age at menopause were observed in either Mexican American or the Other Races subgroups.

Next, we divided the study population into two groups based on survey cycles: 1999–2008 and 2009–2018. The stratified analysis results from both survey cycles remained largely consistent with our prior findings, demonstrating strong generalizability across temporal and demographic strata. Overall, the direction and statistical significance of associations between SDoH factors and age at menopause in subgroup analyses stratified by age, race/ethnicity, and survey cycle were generally consistent with primary findings.

We conducted comprehensive sensitivity analyses through adjustment for smoking status, alcohol consumption, and chronic comorbidities in varying combinations across all study populations (see Table 4, Supplementary Table S4). The results of these additional analyses were largely consistent with those of Model 2 in Table 2, indicating that our conclusions remained statistically significant even after considering other covariates. Specifically, adverse family income and poverty rates, lower educational levels, and marital status (such as being widowed) were associated with an earlier age at menopause. These results further validate the robustness and reliability of our main findings.

**Table 4.** Associations between SDoH and age at menopause: sensitivity analyses with further adjustments using survey-weighted linear regression models, US NHANES 1999–2018 (n = 6083).

SDoH variables	Adjusted for Smoking		Adjusted for Alcohol consumption		Adjusted for Comorbidities	
	β(95% CI)	***P*** ^a^	β(95% CI)	***P*** ^a^	β(95% CI)	***P*** ^a^
**Family PIR**						
* 500% ≤ PIR*	ref		ref		ref	
* 300% ≤ PIR < 500%*	−0.032(−0.577, 0.513)	0.907	−0.026(−0.568, 0.516)	0.925	−0.061(−0.601, 0.479)	0.824
* 100% < PIR < 300%*	−0.848(−1.489, −0.207)	**0.010** **	−0.824(−1.484, −0.165)	**0.015** *	−0.910(−1.556, −0.265)	**0.006** **
* PIR ≤ 100%*	−1.260(−2.066, −0.454)	**0.002** **	−1.251(−2.062, −0.439)	**0.003** **	−1.328(−2.140, −0.517)	**0.002** **
**Education level**						
* College*	ref		ref		ref	
* Some college*	−0.347(−0.855, 0.162)	0.180	−0.386(−0.894, 0.122)	0.135	−0.455(−0.959, 0.049)	0.076
* High school*	−1.195(−1.854, −0.536)	**<0.001** ***	−1.204(−1.847, −0.561)	**<0.001** ***	−1.307(−1.960, −0.653)	**<0.001** ***
* Less than high school*	−1.335(−1.999, −0.672)	**<0.001** ***	−1.340(−2.003, −0.677)	**<0.001** ***	−1.450(−2.113, −0.787)	**<0.001** ***
**Marital status**						
* Married or living with a partner*	ref		ref		ref	
* Never married*	−0.448(−1.188, 0.291)	0.233	−0.471(−1.202, 0.260)	0.204	−0.479(−1.207, 0.249)	0.195
* Widowed*	−1.327(−1.851, −0.803)	**<0.001** ***	−1.358(−1.880, −0.837)	**<0.001** ***	−1.375(−1.902, −0.848)	**<0.001** ***
* Divorced*	−0.060(−0.486, 0.606)	0.828	−0.026(−0.579, 0.526)	0.925	−0.005(−0.546, 0.556)	0.986
* Separated*	−0.227(−1.320, 0.866)	0.682	−0.249(−1.362, 0.864)	0.659	−0.247(−1.348, 0.854)	0.658

Sensitivity analyses were performed based on Model 2, and further adjusted by adding variables including smoking, alcohol consumption, and comorbidities, respectively. Results of adjusted regression coefficients (β), their 95% CIs, and *P* presented with bold values were statistically significant with *P* < 0.05 or *P* < 0.001.

a Asterisks(*) are used to indicate statistical significance levels.

* *P* < 0.05.

** *P* < 0.01.

*** *P* < 0.001.

NHANES, National Health and Nutrition Examination Survey; PIR, family income-to-poverty ratio; SDoH, social determinants of health.

**Figure 3.**
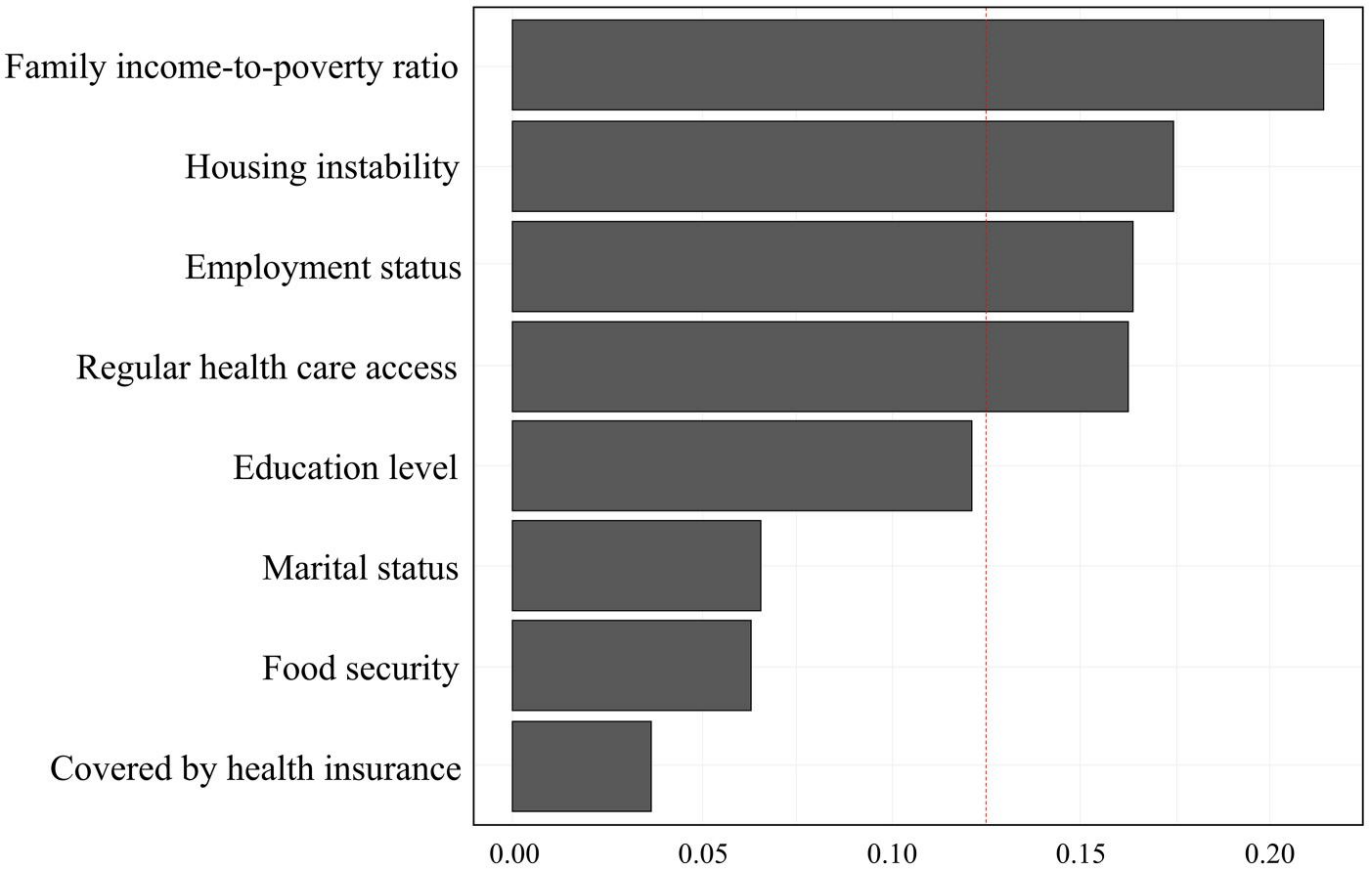
**Weights of each SDoH variable in the WQS model regression indices, US NHANES 1999–2018 (n = 6083).** NHANES, National Health and Nutrition Examination Survey; SDoH, social determinants of health; WQS, weighted quantile sum.

Analysis using a WQS regression model based on decile categorization demonstrated that each 1-unit increase in the composite exposure index of adverse SDoH factors was associated with 3.302 years earlier age at menopause in women (95% CI = −4.129, −2.476, *P* < 0.001; see Table 5 and Fig. 3). We observed that PIR contributed most substantially to the inverse association between SDoH and age at menopause, followed by housing instability, employment status, and regular health care access. In contrast, being covered by health insurance showed the lowest contribution. Estimated weights of each SDoH variable for WQS regression indices are shown in Supplementary Table S5.

**Table 5.** WQS analysis results of SDoH variables with age at menopause, US NHANES 1999–2018 (n = 6083).

Outcome	β(95% CI)	***P*** ^a^
Age at menopause	−3.302 (−4.129, −2.476)	**<0.001** ***

The model was adjusted for age, race/ethnicity, smoking, and alcohol consumption. Results of adjusted regression coefficients (β), their 95% CIs, and *P* presented with bold values were statistically significant with *P* < 0.05 or *P* < 0.001.

a Asterisks(*) are used to indicate statistical significance levels.

* *P* < 0.05.

** *P* < 0.01.

*** *P* < 0.001.

NHANES, National Health and Nutrition Examination Survey; SDoH, social determinants of health; WQS, weighted quantile sum.

## Discussion

### Principal findings

Through the analysis of a nationally representative sample of 6083 women in the USA, we observed that, after adjusting for relevant confounding factors, adverse family PIR, educational level, and marital status (being widowed) were associated with earlier age at menopause. Overall exposure to adverse SDoH factors similarly led to earlier age at menopause.

### Results in the context of what is known

Leone *et al.* (2023) conducted a trend analysis using standard household survey data from 76 countries and found that premature and early menopause are increasing in low- and middle-income countries, particularly in sub-Saharan Africa and South Asia/Southeast Asia. Additionally, an analysis of district-level household survey data in India showed that natural menopause in Indian women is significantly associated with marital disruption, poverty, and socioeconomic factors (Pallikadavath *et al.*, 2016). Another cross-sectional study also found that adverse marital status and lower monthly income were associated with earlier age at natural menopause (Sinha *et al.*, 2021). These findings are consistent with our results, suggesting that economic disadvantage has a significant negative impact on women’s reproductive health.

Regarding educational level, some research results differ from our findings. For example, Sinha *et al.* (2021) found in a study of indigenous populations in North Bengal, India, that women with higher education levels who had reached menarche at age 12 years or older had a significantly earlier ages at menopause compared to women with lower education levels. The varying research outcomes may be attributed to differences in population, geographic location, cultural context, variable definitions, and methods of data analysis. However, most studies are consistent with our conclusions, indicating that lower educational levels are significantly associated with earlier age at menopause (InterLACE Study Team, 2019; Huang *et al.*, 2020). Additionally, a cross-sectional study of Canadian women found that not having a partner, low family income and low educational level were associated with earlier natural menopause, while current employment was associated with later natural menopause (Costanian *et al.*, 2018). This is consistent with our observations.

### Clinical implications

Combining these findings with previous literature, we speculate that economic disadvantage may lead to a chronic state of stress, which can affect hormone levels and reproductive health (Jaillardon and Kaiser, 2023). Similar to economic disadvantage, women with lower education levels often face more social and economic pressures, which can affect reproductive health through various pathways. Additionally, women with lower education levels may lack necessary healthcare knowledge and support, leading to less timely and appropriate management of reproductive health issues, which further impacts their reproductive health. Regarding the impact of adverse marital status, several mechanisms based on existing research may explain these effects. First, individuals with adverse marital status, such as widows, experience higher levels of pro-inflammatory cytokine production and dysregulation of the autonomic nervous and neuroendocrine systems (Fagundes and Wu, 2021), which can affect ovarian function and hormone levels, leading to earlier menopause. Second, after widowhood, women are more likely to experience symptoms of depression and anxiety (Kristiansen *et al.*, 2019; Lin *et al.*, 2019). These psychological issues can affect the secretion of sex hormones and the growth of the endometrium (Wdowiak *et al.*, 2022), thus impacting normal menopause. Finally, after widowhood, women may be more prone to adopting unhealthy lifestyle habits, such as smoking and poor diet (Ding *et al.*, 2021; Pasqualini *et al.*, 2024), and may face greater economic pressure, all of which can exacerbate chronic stress responses, accelerate the aging process, and thereby impair reproductive health. In summary, adverse marital statuses such as widowhood can affect women’s age at menopause through multiple pathways. Understanding these mechanisms can help in developing targeted interventions to improve the reproductive health of affected women.

In our WQS analysis, PIR (weight = 0.214), housing instability (0.175), employment status (0.164), and regular health care access (0.163) collectively formed the core SDoH cluster influencing age at menopause, accounting for 71.6% of the total joint effect. These findings substantiate the implementation of integrated multidimensional interventions targeting economic stability, housing security, employment support, and healthcare access, which would likely yield substantially greater benefits than single-dimensional policy adjustments (e.g. expanding health insurance coverage alone showed only 0.036 weight). Moreover, the gradient distribution of weights (PIR’s weight being ∼6-fold that of health insurance) suggests that material deprivation factors may exert profoundly stronger effects on reproductive aging than institutional safeguards.

In our study, although some subgroups of other SDoH factors, such as employment status, food security, and housing instability, showed significant effects on the outcomes in the initial model (Model 1), these factors lost their significance in the fully adjusted model (Model 2). This may be due to the correlations between these factors and the significant factors (such as PIR, educational level, and marital status), leading to a reduction in their independent effects when controlling for these stronger predictors (even though our correlation heatmap did not show strong correlations, this possibility cannot be entirely ruled out). Additionally, these factors may influence outcomes through indirect pathways. For example, educational level may indirectly affect reproductive health by improving employment opportunities and income levels. Therefore, in the fully adjusted model, when controlling for educational level and other mediating variables (such as PIR), the direct effect of employment opportunities may weaken or disappear. Despite this, these factors remain important in public health policy-making, particularly in promoting socioeconomic equality and improving women’s health.

In our multi-model analyses, the effect sizes of SDoH-menopause age associations showed minimal numerical differences between Model 2 (with race/ethnicity adjustment) and Model 3 (without this adjustment), while maintaining virtually identical statistical significance patterns. From a public health intervention perspective, Model 3 more comprehensively reflects the potential magnitude of health benefits achievable through improving specific SDoH factors within real-world contexts marked by systemic racial disparities. Notably, this unadjusted model may incorporate unmeasured confounding. We therefore propose a dual-interpretation framework: Model 2 provides estimates of ‘direct effects’ controlling for racial confounding, while Model 3 approximates the ‘total effects’ encompassing structurally mediated pathways in social reality. Together, they establish complementary evidence dimensions for policy formulation.

### Research implications

Despite providing valuable insights into how socioeconomic factors influence women’s reproductive health, this study leaves several questions unanswered. First, although we identified the impact of economic disadvantage and lower educational levels on age at menopause, the underlying biological mechanisms still require further exploration. For instance, the specific pathways through which chronic stress and a state of stress affect hormone levels and reproductive health have not been fully clarified. Second, the effects of marital status on women’s reproductive health may differ across different cultural and social contexts. Our study is based primarily on data from specific regions, so more multinational comparative studies are needed to validate these findings and understand their global universality and variability. Additionally, the societal, psychological, and physiological changes following widowhood and their specific impacts on women’s reproductive health need more detailed research, especially regarding long-term effects and potential intervention strategies. Finally, the influence of other SDoH, such as food security and housing instability, on women’s reproductive health also deserves further exploration.

### Strengths and limitations

The strengths of this study lie in the use of a large, nationally representative population to investigate the impact of multiple adverse SDoH on age at menopause, while adjusting for confounding factors such as age, race/ethnicity, smoking, alcohol consumption, and chronic comorbidities. Additionally, this study considered the combined exposure to multiple adverse SDoH to assess their overall impact. More critically, our application of WQS regression transcended the limitations of traditional linear modeling. First, by quantifying composite weights of SDoH factors, it uncovered amplification effects of multidimensional social deprivation. Second, the weight hierarchy established mechanistic priorities, i.e. dominance of the PIR (weight = 0.214), implicating economic stress-related inflammatory pathways as probable core biological mediators. Finally, the weight ratios provide a robust evidence base for policy adjustments addressing health inequalities.

However, the study also has certain limitations. First, many key factors that may influence age at menopause, such as parity, previous hormone use, and exposure to harmful chemicals, were not included due to limitations in model complexity and data availability. These factors can have significant effects on the outcomes and their exclusion may limit the comprehensiveness of our findings. Second, the data for defining various exposure factors and outcome variables primarily relied on self-reported questionnaire responses, which may be subject to participant response and recall biases, potentially introducing certain confounding biases. Finally, due to the cross-sectional design of NHANES, all SDoH data were collected retrospectively after menopause (or at the time of study interview), rather than being dynamically assessed prior to menopause. While certain factors (e.g. educational level) may remain relatively stable during adulthood and are unlikely to have changed substantially between menopause and interview, other variables (e.g. employment status) could have undergone significant changes due to retirement (particularly among women aged ≥65 years) or major life events. Although we attempted to mitigate this bias through age-stratified analyses, residual confounding may persist. Consequently, such cross-sectional data cannot precisely capture premenopausal exposure status, potentially compromising causal inference regarding the association between SDoH factors and age at menopause. Thus, the conclusions drawn should be interpreted with caution, and future investigations should employ longitudinal designs with larger, more representative samples to prospectively evaluate SDoH at critical timepoints throughout the reproductive lifespan (from menarche to menopause). Such rigorous study designs would not only validate our current findings but also more accurately elucidate the complex temporal relationships and underlying biological mechanisms linking SDoH with reproductive aging processes.

## Conclusion

This study, through the analysis of data from 6083 women in the USA, reveals the associations between adverse SDoH and age at menopause. Specifically, adverse PIR, educational level, and marital status (being widowed) are associated with earlier age at menopause. Overall exposure to adverse SDoH factors similarly leads to earlier age at menopause. The PIR contributed most substantially to the inverse association between SDoH and age at menopause. Future research can further investigate the underlying causal relationships between these factors. This study will aid in the development of public health policies for women, providing important evidence to improve women’s health and reduce health inequalities.

## Supplementary Material

hoaf050_Supplementary_Data

## Data Availability

The data underlying this study are publicly available from the National Health and Nutrition Examination Survey (NHANES) database, maintained by the Centers for Disease Control and Prevention (CDC) at https://www.cdc.gov/nchs/nhanes/index.htm.
