# Supplementary material for: Association of social determinants of health and age at menopause: NHANES 1999–2018 observational study
Source: Hum Reprod Open. 2025 Aug 18;2025(3):hoaf050. doi: 10.1093/hropen/hoaf050 (PMC12417080; doi:10.1093/hropen/hoaf050)
Supplement: hoaf050_Supplementary_Data [file hoaf050_supplementary_data.docx]

**Supplementary Data:** **Association of Social Determinants of Health and Age at Menopause: NHANES 1999–2018 Observational Study**

Table of Contents

[**Supplementary Table S1.** Specific definitions of Social Determinants of Health in U.S. NHANES 1999–2018**.4**](#_Table_S1._Specific)

[**Supplementary Table S2.** Associations between Social Determinants of Health and age at menopause: survey-weighted linear regression models stratified by race/ethnicity, U.S. NHANES 1999–2018 (n = 6083)**6**](#_Table_S2._Associations)

[**Supplementary Table S3.** Associations between Social Determinants of Health and age at menopause: survey-weighted linear regression models stratified by survey cycle, U.S. NHANES 1999–2018 (n = 6083)**7**](#_Table_S3._Associations)

[**Supplementary Table S4.** Associations between Social Determinants of Health and age at menopause: supplementary sensitivity analyses with further adjustments using survey-weighted linear regression models, U.S. NHANES 1999–2018 (n = 6083)**8**](#_Table_S4._Associations)

[**Supplementary Table S5.** Estimated weights of each SDoH variable for WQS regression indices, U.S. NHANES 1999–2018 (n = 6083)**9**](#_Table_S5._Estimated)

# **Supplementary Table S1.** Specific definitions of Social Determinants of Health in U.S. NHANES 1999–2018.

| **Domain** | **SDoH variables** | **Criteria for Variable Classification** | **Definitions** |
| --- | --- | --- | --- |
| **Economic Stability** | Employment status | *0: Employed, student, retired*  *1: Not employed* | *Based on questionnaire items OCD150 and OCQ380, participants were asked whether they worked last week and the reasons for not working. Those who clearly stated they were working or identified themselves as students or retirees were classified into one category. Among the remaining individuals, those who clearly indicated they were looking for work or not working were classified into another category.* |
|  | Family income-to-poverty ratio | *1：500% ≤ PIR*  *2：300% ≤ PIR < 500%*  *3：100% < PIR < 300%*  *4：PIR ≤ 100%* | *Based on the demographic variable INDFMPIR, which indicates the ratio of the participant's family income to the poverty level, participants were further categorized.* |
|  | Food security | *1：Full food security (0 affirmative)*  *2：Marginal (1-2 affirmative)*  *3：Low (3-5 affirmative)*  *4：Very low (6-10 affirmative)* | *Based on questionnaire items FSDAD/ADFDSEC, participants answered 10 FSSM questions. According to the number of affirmative responses to these questions, participants were categorized into the following classes: 1.Adult Full Food Security: No affirmative responses to any of these items. 2.Adult Marginal Food Security: 1-2 affirmative responses. 3.Adult Low Food Security: 3-5 affirmative responses. 4.Adult Very Low Food Security: 6-10 affirmative responses. The questions included: 1.Whether they were worried that the food would run out before they got money to buy more during the past 12 months. 2.Whether the food they bought just didn’t last, and they didn’t have money to get more. 3.Whether they couldn’t afford to eat balanced meals. 4.Whether they had ever cut the size of meals or skipped meals because there wasn’t enough money for food. 5.How often they had ever cut the size of meals or skipped meals because there wasn’t enough money for food. 6.Whether they had ever eaten less than they felt they should because there wasn’t enough money to buy food. 7.Whether they were ever hungry but didn’t eat because they couldn’t afford enough food. 8.Whether they had lost weight because they didn’t have enough money for food. 9.Whether they had ever gone a whole day without eating because there wasn’t enough money for food. 10.How often they had ever gone a whole day without eating because there wasn’t enough money for food.* |
| **Education Access and Quality** | Education level | *1：College*  *2：Some college*  *3：Highschool*  *4：Less than high school* | *Based on the demographic variable DMDEDUC2, which indicates the highest level of education completed or the highest degree received by the participant.* |
| **Health Care Access and Quality** | Regular health care access | *0：Routine place to go for healthcare*  *1：No routine place, or ER/hospital/other* | *Based on questionnaire items HUQ030 and HUQ041/HUQ040, participants were asked whether they have a usual place to go when sick or needing health advice, and what type of healthcare facility they usually visit. Those who responded that they do not have a regular place to go or that they usually go to a hospital emergency room were classified into one category. The remaining participants were classified into another category.* |
|  | Covered by health insurance | *1：Private insurance*  *2：Government insurance*  *3：No insurance* | *Based on questionnaire items HIQ011/HID010 and HIQ031A/HID030A, participants were asked whether they have health insurance coverage and whether they have private insurance coverage. Those who reported having health insurance but no private insurance were considered to have government insurance coverage.* |
| **Neighborhood and Built Environment** | Housing instability | *1：Own home*  *2：Rent home*  *3：Other arrangement* | *Based on questionnaire item HOQ065, participants were asked whether the housing is owned outright, being purchased, rented, or occupied through other arrangements by themselves or other family members. Those who responded that the housing is owned or being purchased were considered to own their home.* |
| **Social and Community Context** | Marital status | *1：Married or living with a partner*  *2：Never married*  *3：Widowed*  *4：Divorced*  *5：Separated* | *Based on the demographic variable DMDMARTL, which indicates the marital status of the participant.* |

Footnotes: The definition of SDoH variables in this study specifically referred to the methods outlined in two previous publications(Bundy et al., 2023; Liang et al., 2024), adopting SDoH variables under the five domains of "Healthy People 2030" (Economic Stability, Education Access and Quality, Health Care Access and Quality, Neighborhood and Built Environment, and Social and Community Context).

Bundy JD, Mills KT, He H, LaVeist TA, Ferdinand KC, Chen J, He J. Social determinants of health and premature death among adults in the USA from 1999 to 2018: a national cohort study. Lancet Public Health 2023;8:e422-e431.

Liang JH, Liu ML, Pu YQ, Huang S, Jiang N, Bao WW, Hu LX, Zhang YS, Gui ZH, Pu XY et al. Cumulative inequality in social determinants of health in relation to depression symptom: An analysis of nationwide cross-sectional data from U.S. NHANES 2005-2018. Psychiatry Res 2024;336:115894.

Abbreviations: NHANES, National Health and Nutrition Examination Survey; PIR, family income-to-poverty ratio; SDoH, Social Determinants of Health

# **Supplementary Table S2**. Associations between Social Determinants of Health and age at menopause: survey-weighted linear regression models stratified by race/ethnicity, U.S. NHANES 1999–2018 (n = 6083)

| **SDoH variables** | **Mexican American(n=985)** | | **Non-Hispanic Black(n=1077)** | | **Non-Hispanic White(n=2952)** | | **Other Races(n=1069)** | |
| --- | --- | --- | --- | --- | --- | --- | --- | --- |
|  | **β(95% CI)** | ***P***^a^ | **β(95% CI)** | ***P***^a^ | **β(95% CI)** | ***P***^a^ | **β(95% CI)** | ***P***^a^ |
| **Family income-to-poverty ratio** |  |  |  |  |  |  |  |  |
| *500% ≤ PIR* | ref |  | ref |  | ref |  | ref |  |
| *300% ≤ PIR < 500%* | −1.036(−2.765, 0.693) | 0.233 | 1.191(−0.542, 2.924) | 0.175 | −0.057(−0.690, 0.575) | 0.858 | −0.426(−1.653, 0.801) | 0.491 |
| *100% < PIR < 300%* | −1.120(−2.809, 0.568) | 0.188 | −0.702(−2.336, 0.933) | 0.394 | −0.891(−1.649, −0.134) | **0.021*** | −0.094(−1.387, 1.199) | 0.886 |
| *PIR ≤ 100%* | −1.531(−3.298, 0.236) | 0.088 | −0.067(−2.267, 2.133) | 0.952 | −1.736(−2.818, −0.653) | **0.002**** | −0.282(−2.073, 1.508) | 0.754 |
| **Education level** |  |  |  |  |  |  |  |  |
| *College* | ref |  | ref |  | ref |  | ref |  |
| *Some college* | −0.543(−2.527, 1.441) | 0.583 | −1.236(−2.369, −0.102) | **0.033*** | −0.299(−0.885, 0.286) | 0.314 | −0.594(−1.744, 0.555) | 0.306 |
| *Highschool* | −0.156(−1.876, 2.188) | 0.878 | −0.385(−1.569, 0.799) | 0.518 | −1.472(−2.236, −0.708) | **<0.001***** | −0.611(−1.748, 0.526) | 0.288 |
| *Less than high school* | −0.316(−2.090, 1.458) | 0.721 | −1.634(−3.024, −0.245) | **0.022*** | −1.699(−2.565, −0.833) | **<0.001***** | −0.356(−1.600, 0.889) | 0.571 |
| **Marital status** |  |  |  |  |  |  |  |  |
| *Married or living with a partner* | ref |  | ref |  | ref |  | ref |  |
| *Never married* | −0.047(−2.066, 1.973) | 0.963 | 0.164(−1.171, 1.498) | 0.807 | −0.554(−1.672, 0.563) | 0.328 | −1.306(−2.789, 0.178) | 0.084 |
| *Widowed* | −0.651(−1.869, 0.566) | 0.286 | −0.870(−2.139, 0.399) | 0.175 | −1.422(−2.040, −0.803) | **<0.001***** | −1.377(−2.889, 0.136) | 0.074 |
| *Divorced* | −0.275(−1.451, 0.901) | 0.640 | 0.873(−0.072, 1.818) | 0.070 | −0.084(−0.798, 0.630) | 0.817 | −0.291(−1.596, 1.014) | 0.658 |
| *Separated* | −0.029(−1.328, 1.387) | 0.965 | −0.363(−2.473, 1.747) | 0.732 | −0.110(−2.295, 2.516) | 0.928 | −0.741(−2.352, 0.869) | 0.362 |

Footnotes: For each Social Determinant of Health (SDoH) variable within the racial subgroups, the polynomial linear model was adjusted for age and the other 7 SDoH variables. The results show the adjusted regression coefficients (β), their 95% confidence intervals (95% CI), and P. Bold values indicate statistical significance (P< 0.05 or P< 0.001).

^a^ Asterisks (*) are used to indicate statistical significance levels: *P < 0.05, **P < 0.01, ***P < 0.001

Abbreviations: CI, Confidence interval; NHANES, National Health and Nutrition Examination Survey; PIR, family income-to-poverty ratio; SDoH, Social Determinants of Health

# Supplementary Table S3. Associations between Social Determinants of Health and age at menopause: survey-weighted linear regression models stratified by survey cycle, U.S. NHANES 1999–2018 (n = 6083)

| **SDoH variables** | **1999–2008(n=2682)** | | **2009–2018(n=3401)** | |
| --- | --- | --- | --- | --- |
|  | **β(95% CI)** | ***P***^a^ | **β(95% CI)** | ***P***^a^ |
| **Family income-to-poverty ratio** |  |  |  |  |
| *500% ≤ PIR* | ref |  | ref |  |
| *300% ≤ PIR < 500%* | 0.463(−0.256, 1.182) | 0.202 | −0.384(−1.127, 0.358) | 0.304 |
| *100% < PIR < 300%* | −1.214(−2.138, −0.290) | **0.011*** | −0.648(−1.523, 0.227) | 0.144 |
| *PIR ≤ 100%* | −1.132(−2.274, 0.009) | 0.052 | −1.442(−2.570, −0.313) | **0.013*** |
| **Education level** |  |  |  |  |
| *College* | ref |  | ref |  |
| *Some college* | −0.686(−1.413, 0.041) | 0.064 | −0.225(−0.901, 0.451) | 0.508 |
| *Highschool* | −1.478(−2.333, −0.622) | **0.001**** | −1.103(−2.055, −0.151) | **0.024*** |
| *Less than high school* | −1.567(−2.593, −0.541) | **0.003**** | −1.219(−2.111, −0.327) | **0.008**** |
| **Marital status** |  |  |  |  |
| *Married or living with a partner* | ref |  | ref |  |
| *Never married* | −0.987(−2.180, 0.207) | 0.103 | −0.167(−1.115, 0.781) | 0.725 |
| *Widowed* | −1.585(−2.395, −0.776) | **<0.001***** | −1.164(−1.848, −0.481) | **0.001**** |
| *Divorced* | −0.044(−0.867, 0.779) | 0.916 | −0.003(−0.740, 0.746) | 0.994 |
| *Separated* | −0.315(−1.814, 1.183) | 0.674 | −0.263(−1.831, 1.305) | 0.738 |

Footnotes: For each Social Determinant of Health (SDoH) variable within the year subgroups, the polynomial linear model was adjusted for age and race/ethnicity, as well as the other 7 SDoH variables. The results show the adjusted regression coefficients (β), their 95% confidence intervals (95% CI), and P. Bold values indicate statistical significance (P< 0.05 or P< 0.001).

^a^ Asterisks (*) are used to indicate statistical significance levels: *P < 0.05, **P < 0.01, ***P < 0.001

Abbreviations: CI, Confidence interval; NHANES, National Health and Nutrition Examination Survey; PIR, family income-to-poverty ratio; SDoH, Social Determinants of Health

# Supplementary Table S4. Associations between Social Determinants of Health and age at menopause: supplementary sensitivity analyses with further adjustments using survey-weighted linear regression models, U.S. NHANES 1999–2018 (n = 6083)

| **Adjusted for** | **Smoking+** **Alcohol consumption** | | **Alcohol consumption+** **Comorbidities** | | **Smoking+** **Comorbidities** | | **Smoking+** **Alcohol consumption+** **Comorbidities** | |
| --- | --- | --- | --- | --- | --- | --- | --- | --- |
| **SDoH variables** | **β(95% CI)** | ***P***^a^ | **β(95% CI)** | ***P***^a^ | **β(95% CI)** | ***P***^a^ | **β(95% CI)** | ***P***^a^ |
| **Family income-to-poverty ratio** |  |  |  |  |  |  |  |  |
| *500% ≤ PIR* | ref |  | ref |  | ref |  | ref |  |
| *300% ≤ PIR < 500%* | 0.006(−0.541, 0.552) | 0.984 | −0.038(−0.579, 0.504) | 0.890 | −0.045(−0.589, 0.499) | 0.871 | −0.007(−0.553, 0.540) | 0.981 |
| *100% < PIR < 300%* | −0.762(−1.415, −0.109) | **0.023*** | −0.857(−1.514, −0.200) | **0.011*** | −0.882(−1.520, −0.244) | **0.007**** | −0.796(−1.446, −0.145) | **0.017*** |
| *PIR ≤ 100%* | −1.183(−1.990, −0.375) | **0.004**** | −1.282(−2.096, −0.469) | **0.002**** | −1.292(−2.101, −0.484) | **0.002**** | −1.215(−2.025, −0.405) | **0.004**** |
| **Education level** |  |  |  |  |  |  |  |  |
| *College* | ref |  | ref |  | ref |  | ref |  |
| *Some college* | −0.292(−0.808, 0.224) | 0.265 | −0.429(−0.937, 0.078) | 0.097 | −0.390(−0.899, 0.118) | 0.131 | −0.336(−0.852, 0.179) | 0.199 |
| *Highschool* | −1.093(−1.743, −0.443) | **0.001**** | −1.249(−1.893, −0.604) | **<0.001***** | −1.241(−1.901, −0.580) | **<0.001***** | −1.138(−1.790, −0.487) | **<0.001***** |
| *Less than high school* | −1.226(−1.895, −0.557) | **<0.001***** | −1.387(−2.054, −0.721) | **<0.001***** | −1.383(−2.050, −0.717) | **<0.001***** | −1.273(−1.946, −0.601) | **<0.001***** |
| **Marital status** |  |  |  |  |  |  |  |  |
| *Married or living with a partner* | ref |  | ref |  | ref |  | ref |  |
| *Never married* | −0.421(−1.161, 0.319) | 0.263 | −0.466(−1.193, 0.261) | 0.207 | −0.443(−1.179, 0.293) | 0.236 | −0.414(−1.151, 0.322) | 0.268 |
| *Widowed* | −1.311(−1.831, −0.791) | **<0.001***** | −1.370(−1.895, −0.845) | **<0.001***** | −1.338(−1.866, −0.811) | **<0.001***** | −1.323(−1.846, −0.799) | **<0.001***** |
| *Divorced* | 0.036(−0.511, 0.583) | 0.897 | −0.021(−0.573, 0.530) | 0.939 | −0.066(−0.478, 0.610) | 0.810 | −0.042(−0.504, 0.588) | 0.879 |
| *Separated* | −0.242(−1.340, 0.855) | 0.663 | −0.260(−1.366, 0.847) | 0.643 | −0.237(−1.324, 0.851) | 0.667 | −0.253(−1.344, 0.838) | 0.647 |

Footnotes: Sensitivity analyses were performed based on Model 2 and further adjusted by adding different combinations of variables including smoking, alcohol consumption, and comorbidities, to assess the robustness of the model. Results of adjusted regression coefficients (β), their 95% confidence intervals (95% CI), and P presented with bold values were statistically significant with P < 0.05 or P < 0.001.

^a^ Asterisks (*) are used to indicate statistical significance levels: *P < 0.05, **P < 0.01, ***P < 0.001

Abbreviations: CI, Confidence interval; NHANES, National Health and Nutrition Examination Survey; PIR, family income-to-poverty ratio; SDoH, Social Determinants of Health

# Supplementary Table S5. Estimated weights of each SDoH variable for WQS regression indices, U.S. NHANES 1999–2018 (n = 6083)

| **SDoH variable** | **Estimated weight** |
| --- | --- |
| Family income-to-poverty ratio | 0.214 |
| Housing instability | 0.175 |
| Employment status | 0.164 |
| Regular health care access | 0.163 |
| Education level | 0.121 |
| Marital status | 0.066 |
| Food security | 0.062 |
| Covered by health insurance | 0.036 |

Footnotes: The model was adjusted for age, race/ethnicity, smoking and alcohol consumption.

Abbreviations: NHANES, National Health and Nutrition Examination Survey; SDoH, Social Determinants of Health; WQS, Weighted Quantile Sum
